# Rapidly progressing, fatal and acute promyelocytic leukaemia that initially manifested as a painful third molar: a case report

**DOI:** 10.1186/1752-1947-3-102

**Published:** 2009-11-03

**Authors:** Juan A Suárez-Cuenca, José L Arellano-Sánchez, Aldo A Scherling-Ocampo, Gerardo Sánchez-Hernández, David Pérez-Guevara, Juan R Chalapud-Revelo

**Affiliations:** 1 Department of Internal Medicine, Ticomán General Hospital, SSDF Mexico City, Mexico; 2 Xoco General Hospital, SSDF Av México Coyoacán s/n, esq Bruno Traven, Mexico City, Mexico; 3 Cellular Biology Department (Postgraduate Program in Biomedical Science), Institute of Cellular Physiology, Circuito Exterior s/n Ciudad Universitaria, Mexico City, Mexico; 4 Maxillofacial Surgery Department (Division of Research and Postgraduate Studies), Faculty of Dentistry, Av Institutos s/n Ciudad Universitaria, Mexico City, Mexico; 5 Haematology Department, National Cancer Institute, Av San Fernando, Mexico City, Mexico

## Abstract

**Introduction:**

Acute promyelocytic leukaemia, an uncommon and devastating subtype of leukaemia, is highly prevalent in Latin American populations. The disease may be detected by a dentist since oral signs are often the initial manifestation. However, despite several cases describing oral manifestations of acute promyelocytic leukaemia and genetic analysis, reports of acute promyelocytic leukaemia in Hispanic populations are scarce. The identification of third molar pain as an initial clinical manifestation is also uncommon. This is the first known case involving these particular features.

**Case presentation:**

A 24-year-old Latin American man without relevant antecedents consulted a dentist for pain in his third molar. After two dental extractions, the patient experienced increased pain, poor healing, jaw enlargement and bleeding. A physical examination later revealed that the patient had pallor, jaw enlargement, ecchymoses and gingival haemorrhage. Laboratory findings showed pancytopaenia, delayed coagulation times, hypoalbuminaemia and elevated lactate dehydrogenase. Splenomegaly was detected on ultrasonography. Peripheral blood and bone marrow analyses revealed a hypercellular infiltrate of atypical promyelocytic cells. Cytogenetic analysis showing genetic translocation t(15;17) further confirmed acute promyelocytic leukaemia. Despite early chemotherapy, the patient died within one week due to intracranial bleeding secondary to disseminated intravascular coagulation.

**Conclusion:**

The description of this unusual presentation of acute promyelocytic leukaemia, the diagnostic difficulties and the fatal outcome are particularly directed toward dental surgery practitioners to emphasise the importance of clinical assessment and preoperative evaluation as a minimal clinically-oriented routine. This case may also be of particular interest to haematologists, since the patient's cytogenetic analysis, clinical course and therapeutic response are well documented.

## Introduction

Up to 65% of patients with acute leukaemia consult a dentist due to oral manifestations, or the disease is detected from suggestive findings during periodontal and/or physical examination. According to reports, the most common findings in the oral cavity include gingival enlargement, local abnormal colour or gingival haemorrhage, petechiae, ecchymoses, mucosal ulceration, paresthesia and/or oral infections [1]. These clinical manifestations are consequences of gingival infiltration and abnormal proliferative neoplastic white blood cells that affect the normal production of erythrocytes, leukocytes and platelets. Toothache as an initial clinical manifestation of acute leukaemia without accompanying oral or systemic manifestation is very uncommon [2,3].

Acute promyelocytic leukaemia (M3-APL) is a malignant subtype of acute myeloid leukaemia (AML), comprising approximately 8 to 13% of reported cases of leukaemia. Prevalence is especially high among Hispanic populations and among younger patients [4]. In most cases, chromosomal abnormality from genetic translocation t(15;17) that leads to leukaemic transformation can be seen by cytogenetics [5]. Oral symptoms in M3-APL are similar to those found in other leukaemias. Takagi et al. [6] described oral manifestations in 16 patients with spontaneous gingival bleeding, post-oral surgery bleeding and gingival swelling as the most common symptoms of M3-APL. Half of these patients consulted a dentist during an early stage of the disease.

The clinical outcome of M3-APL is characterised by bleeding disorders secondary to disseminated intravascular coagulation (DIC). This may account for the worst features associated with leukaemia since it causes a fulminant disorder that primarily affects young people. The effects are devastating on an individual's life and causes death for a large number of patients during the initial phases of treatment. However, M3-APL is the most treatable of AMLs if early diagnosis and treatment are performed [7].

## Case presentation

A 24-year-old, obese, Latin American man with a history of measles, scarlet fever, and appendectomy, and taking no medications, consulted a private odontological care centre because of two days of toothache on the right side of his mouth. Evaluated as a common painful third molar, surgical extraction was attempted, but resulted in partial extraction due to the dentist's inexperience. The patient experienced mild haemorrhage and pain which was controlled by conventional haemostatic measures and analgesia. A prophylactic antibiotic was prescribed to the patient, and a later appointment was scheduled for the extraction of the residual tooth.

On the following day, the patient continued to have mild haemorrhaging and local swelling. The same supportive measures were prescribed since these were considered normal outcomes of a traumatic procedure. On the third day after surgery, persistent pain accompanied by malaise, moderate haemorrhaging and progressive local swelling prompted hospital care, where management included conventional haemostatic measures, analgesic, one-day hospital surveillance and early discharge. No laboratory analysis was ordered.

The next day, the patient complained of mild local pain, accompanied by trismus, minor local bleeding and clots, ecchymoses and mild jaw enlargement. Surgical extraction of the tooth residues and wound closure were performed, without apparent hemorrhagic complications. The dentist assumed that the patient had a coagulation disorder, based on such slow healing, and recommended a blood test and medical evaluation at the hospital because the private odontological clinic lacked the necessary laboratory resources. That same day, the patient experienced exacerbated pain, poor response to analgesia and continued local bleeding, which required emergency hospital care and a stay in the internal medicine department.

**Figure 1:**
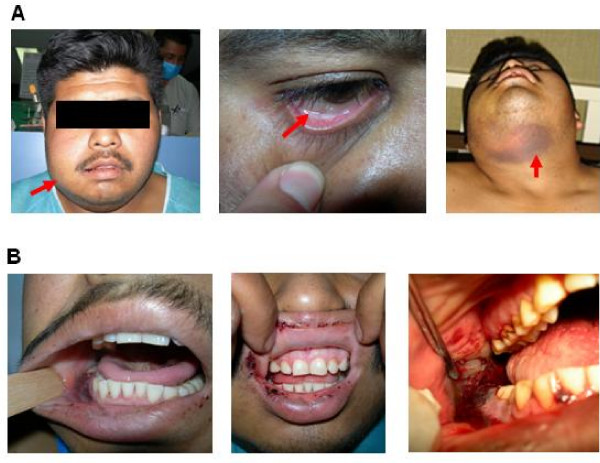
**Clinical appearance of the patient after second dental procedure**. Upon hospital admission, the patient exhibited jaw enlargement, conjunctival pallor and ecchymoses (panel A, left, middle and right, respectively). Oral cavity examination showed gingival damage with active gingival haemorrhage from surgery (panel B).

Physical examination revealed that the patient had pallor, right-sided jaw enlargement, ecchymoses (Figure 1, panel A) and an oral cavity with active gingival haemorrhage from surgery (Figure 1, panel B). There was no gingival enlargement, local abnormal colour, or clinical hepatosplenomegaly and/or lymphadenopathy.

The patient's complete blood count (CBC) (Table 1) showed pancytopaenia with differential count of neutrophils 0.489 × 10^9^/L (31.5%), lymphocytes 0.924 × 10^9^/L (59.5%), monocytes 0.122 × 10^9^/L (7.84%), eosinophils 0.006 × 10^9^/L (0.38%), and basophils 0.012 × 10^9^/L (0.76%). The patient had normocytic normochromic anaemia (Hb 107 g/L, MCV 84fL, MCH 29 pg and MCHC 351 g/L), thrombocytopenia (platelets 6.3 × 10^9^/L) and abnormal coagulation times (prothrombin time (PT) 16 s with control sample 11 s, INR 1.43, activated partial tissue thromboplastin (aPTT) 39 s with control sample 32 s). Additional laboratory data revealed dyslipidaemia, hypoalbuminaemia and high levels of lactate dehydrogenase. An abdominal ultrasound revealed mild splenomegaly.

**Table 1:** Complete blood count and blood chemistry

Complete Blood Count	
**Test**	**Value**

**White Blood Cells**	1.5 × 10^9^/L

**Red Blood Cells**	3.6 × 10^12^/L

**Platelets**	6.3 × 10^9^/L

**Haemoglobin**	107.0 g/L

**Haematocrit**	0.30

**Glucose**	6.9 mmol/L

**Blood Biochemistry**	

**Test**	**Value**

**BUN**	12.8 mmol/L

**Creatinine**	159.1 μmol/L

**Uric acid**	422.31 μmol/L

**Calcium**	2.14 mmol/

**Magnesium**	1.03 mmol/L

**Cholesterol**	3.59 mmol/L

**C-HDL**	0.21 mmol/L

**C-LDL**	1.91 mmol/L

**Triglycerides**	3.21 mmol/L

**Total bilirubin**	9.06 μmol/L

**Indirect bilirubin**	6.33 μmol/L

**Total protein**	86 g/L

**Globulin**	30 mg/L

**Albumin**	27 g/L

**Aspartate aminotransferase**	30 U/L

**Alanine aminotransferase**	31 U/L

**Alkaline phosphatase**	75 U/L

**Gamma-glutamyl transferase**	82 U/L

**Lactate dehydrogenase (diluted)**	742 U/L

BUN, Blood Urea Nitrogen; C-HDL, High density lipoprotein cholesterol; C-LDL, Low density lipoprotein cholesterol.

**Figure 2:**
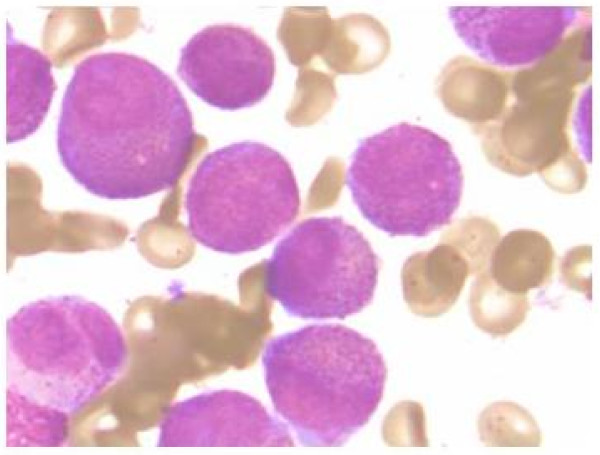
**Histological features of acute promyelocytic leukaemia (M3 subtype from FAB)**. Bone marrow aspirate shows neoplastic promyelocytes, with abnormally coarse and numerous azurophilic granules (1000×).

**Figure 3:**
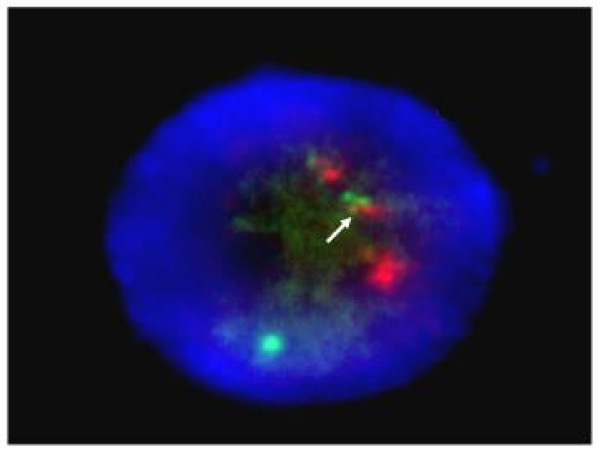
**Cytogenetic analysis reveals chromosome translocation t(15;17)**. Fluorescence in situ hybridisation (FISH) of abnormal promyelocytic cells with a translocation of chromosome 15 and 17 (nucleus in blue, PML gene in red, RARα in green and arrow shows fused signals at translocation 15 and 17).

During a short stay of three days in the internal medicine department, a peripheral blood smear revealed a 6% blast cell content with Auer rods and promyelocytic cells. The patient was referred to the National Cancer Institute, where M3-APL was diagnosed based on bone marrow samples, with hypercellularity and 80% neoplastic promyelocytes (Figure 2). The findings were subsequently confirmed by fluorescence in situ hybridisation (FISH) for chromosomal translocation t(15;17) (Figure 3).

Supportive measures were established based on the patient's stay at the internal medicine department. The patient experienced transfusion-associated fever that occurred after he was transfused with erythrocytes and platelets. Thrombocytopenia was refractory to platelet transfusion and he maintained platelet counts between 6.3 × 10^9^/L and 30 × 10^9^/L. The patient further developed neutropaenia and fever, and the protocol for a high risk of opportunistic infection included isolation and antibiotics (ceftriaxone, metronidazole and fluconazole). At the National Cancer Institute, specific treatment was started immediately after diagnosis. His induction therapy included ATRA (all-trans retinoic acid) and daunorubicin. Laboratory analyses showed platelets at 8 × 10^9^/L, delayed coagulation times and a fibrinogen level of 43 mg/dl (normal range 159 to 317). Further supportive measures included cryoprecipitates in addition to fresh frozen plasma (15 mg/kg q.i.d.) when the patient's fibrinogen level was lower than 100 mg/dl, and platelet transfusion when his platelet count was below 50 × 10^9^/L. His CBC and coagulation parameters (PT, aPTT, thrombin time and fibrinogen levels) were monitored daily.

Despite early specific treatment, the patient suffered generalised seizures secondary to intracranial bleeding caused by DIC, which was confirmed by delayed coagulation times, low serum fibrinogen, high fibrin degradation products and D-dimer. The patient died after four days of hemorrhagic complications.

## Discussion

Oral manifestations of many haematological diseases are clinically similar to locally-occurring lesions. For this reason, a specific diagnosis of blood dyscrasia is difficult, if not impossible, to establish on the basis of oral findings alone. Commonly, the dentist is the first professional in contact with the patient and has the best chance to detect special cases by means of an accurate medical history, a recording of systemic disease-oriented information and an identification of signs that are relevant to the patient's current problem.

Toothache is a common symptom in oral medicine and possible sources include local diseases like tooth infection, decay, nerve irritation, repetitive motions and any injury that bruises the tooth. Non-dental causes include acute ulceration of the gingiva or soft tissues, pericoronitis, dry socket, trigeminal neuralgia, sinusitis, otitis media, mastoiditis, temporomandibular joint pain radiation, and, occasionally, systemic diseases. In this case, third molar pain was identified and treated by surgical extraction. However, a few special considerations may be appropriate. During routine practice of dental extraction, preoperative assessment commonly includes a brief clinical history with details about the outcome of previous dental procedures, including the duration of bleeding after extraction and treatment, previous hemorrhagic episodes, outcomes from other surgical procedures or injuries, family history, drugs or medications, identification or hospital cards and physical findings. Laboratory blood analyses are only performed if the abovementioned items are suspicious. The lack of suggestive medical and/or family history or symptoms in the present case made the diagnosis of this haematological disease unlikely.

Nevertheless, the physical examination and outcomes revealed the manifestations of the underlying disease: a) patient appearance with pallor guided to anaemia, b) poor tissue healing was secondary to leucopoenia, and c) excessive bleeding after extraction is commonly an acute leukaemia-associated symptom due to thrombocytopenia and delayed coagulation times [1,8]. In this case, the excessive bleeding was underestimated. If CBC and coagulation tests had been ordered by the dentist or during the one-day hospital surveillance, it would have led to an earlier diagnosis and treatment that could have possibly lead to a better outcome. However, in the absence of supporting data, hemorrhagic manifestations are easily misinterpreted as a consequence of an imperfect surgical technique and local damage instead of a haematological disease. In the case of thrombocytopenia-related gingival bleeding, local treatment is recommended. Once bleeding is controlled, an early reference to a haematologist or internist should occur if there is any suspicion of a possible haematological disease.

The patient was referred to our hospital without any diagnostic consideration or laboratory data. Upon admission to the hospital, the patient's CBC indicated that a haematological disease was a possibility. Pancytopaenia clearly supported a haematological disorder, while normocytic normochromic anaemia, thrombocytopenia, mild splenomegaly, and abnormally elevated levels of LDH reasonably ruled out other causes of pancytopaenia and suggested bone marrow involvement with a neoplastic origin as a likely cause. A diagnostic protocol and initial supportive measures were rapidly established at the internal medicine department. In collaboration with the National Cancer Institute, the diagnosis of M3-APL was made based on the identification of abnormally excessive haematopoietic cells, namely promyelocytes, in the peripheral blood and bone marrow. Chromosomal translocation t(15;17) was revealed by FISH to further confirm the diagnosis.

M3-APL is considered a special variant for its particular biology, severe manifestations, haematological complications and rapid fatal course. Conversely, M3-APL is currently the AML with the highest rate of favourable response to therapy, especially at an early stage. Morphologically, the bone marrow is effaced by heavily granulated cells with folded and twisted nuclei. In most cases, reciprocal translocation t(15;17) of the PML gene on chromosome 15q22 and the RARα gene on chromosome 17, can be seen cytogenetically [5]. Its demonstration is important because the molecular rearrangement is crucial for leukaemogenesis and treatment. Furthermore, demonstration of t(15;17) or PML/RARα gene fusion is a mandatory requirement for confirming the diagnosis of M3-APL and has relevant prognostic implications [9,10]. Cytochemistry and immunophenotyping provide additional characteristics of M3-APL, and, although not essential for diagnosis, yield to desirable analyses in most cases.

M3-APL frequently affects younger, obese and Latin American populations [4,11]. Patients typically display symptoms associated with cytopaenias. Oral symptoms of M3-APL are similar to those found in other leukaemias, including spontaneous gingival bleeding, post-oral surgery bleeding and gingival swelling [6]. The outcome is characterised by bleeding disorders, which are the main causes of death within the first ten days after onset [12]. Most of these features were true for this case (young Hispanic man with post-extraction toothache, pallor, gingival haemorrhage, ecchymoses, jaw enlargement, pancytopaenia, evidence of neoplastic haematopoietic cells and fatal hemorrhagic complications). Moreover, life-threatening coagulopathy accompanying M3-APL is relatively common (80% of cases at the time of diagnosis) and is ascribed to either primary fibrinolysis or DIC, resulting from abnormal promyelocytes lysis and release of procoagulant mediators [13,14].

Early recognition of M3-APL is important because current therapy of ATRA combined with chemotherapy or arsenic trioxide regimens results in 70 to 80% of patient survival and disease-free outcome after five years. Despite the favourable response rate, patients with M3-APL and bleeding disorders, especially with intracranial bleeding, have an unfavourable prognosis. Peri-induction mortality is significant, reaching around 50%, according to literature reports [13,15].

## Conclusion

Periodontal signs are commonly the first clinical manifestations of AML, including M3-APL. This case emphasises the importance of performing a detailed periodontal history and also obtaining relevant medical information, adequate interpretation of physical findings and following established protocols. Furthermore, it highlights the relevance of a systematic clinical assessment by dental surgeons by ordering pertinent laboratory tests and making early references to haematologists and/or internists if any suspicions arise from the preoperative assessment.

## Abbreviations

M3-APL: Acute Promyelocytic Leukaemia; AML: Acute Myeloid Leukaemia; DIC: disseminated intravascular coagulation; CBC: Complete Blood Count; aPTT: Partial Tissue Thromboplastin; FISH: Fluorescence in situ hybridisation; ATRA: All-trans Retinoic Acid.

## Consent

Written informed consent was obtained from the patient's family for publication of this case report and any accompanying images. A copy of the written consent is available for review by the Editor-in-Chief of this journal.

## Competing interests

The authors declare that they have no competing interests.

## Authors' contributions

JASC participated in the clinical care and follow up of the patient, data collection, biomedical discussion and manuscript elaboration. JLAS and AASO substantially contributed to literature review, data collection and analysis, conception and drafting of the manuscript. DPG was involved in the acquisition of photo and initial dental care of the patient. JRCR was responsible for haematology-oncology care. He also contributed in drafting the manuscript, pathology and cytogenetic data collection. GSH made substantial contributions to the conception and design of the manuscript, data analysis and interpretation.

## References

[B1] Stafford R Sonis S Lockart P Sonis A Oral pathoses as diagnostic indicators in leukemia Oral Surg Oral Med Oral Pathol 1980 50 134 139 10.1016/0030-4220(80)90200-5 6967202

[B2] Gordon MR O'Neal RB Woodyard SG A variation from classic oral manifestations associated with acute myeloblastic leukemia J Periodontol 1985 56 285 287 3859635 10.1902/jop.1985.56.5.285

[B3] Kanas RJ Jensen JL DeBoom GW Painful, nonhealing, tooth extraction socket J Am Dent Assoc 1986 113 441 442 3463617 10.14219/jada.archive.1986.0194

[B4] Douer D Preston-Martin S Chang E Nichols PW Watkins KJ Levine AM High frequency of acute promyelocytic leukemia among Latinos with acute myeloid leukemia Blood 1996 87 308 313 8547657

[B5] Lo Coco Diveiro D Falini B Biondi A Nervi C Pelicci PG Genetic diagnosis and molecular monitoring in the management of acute promyelocytic leukemia Blood 1999 94 12 22 10381493

[B6] Takagi M Sakota Y Ishikawa G Kamiyama R Nakajima T Nomura T Oral manifestations of acute promyelocytic leukemia J Oral Surg 1978 36 589 593 277651

[B7] Soignet SL Maslak PG Acute Promyelocytic Leukemia Wintrobe's Clinical Hematology 2004 2 Philadelphia: Lippincott Williams & Wilkins 2191 2205

[B8] Barret AP Gingival Lesions in Leukemia J Periodontol 1984 55 585 588 6387081 10.1902/jop.1984.55.10.585

[B9] Larson RA Dondo K Vardiman JW Butler AE Golomb HM Rowley JD Evidence for a 15;17 translocation in every patient with acute promyelocytic leukaemia Am J Med 1984 76 827 841 10.1016/0002-9343(84)90994-X 6586073

[B10] Gupta V Yib QL Clinico-biological features and prognostic significance of PML/RARalpha isoforms in adult patients with acute promyelocytic leukemia treated with all trans retinoic acid (ATRA) and chemotherapy Leuk Lymphoma 2004 45 469 480 10.1080/10428190310001617295 15160908

[B11] Estey E Thall P Kantarjian H Pierce S Kornblau S Keating M Association between increased body mass index and a diagnosis of acute promyelocytic leukemia in patients with acute myeloid leukemia Leukemia 1997 11 1661 1664 10.1038/sj.leu.2400783 9324286

[B12] Lowenberg B Griffin JD Tallman MS Acute myeloid leukemia and acute promyelocytic leukemia Hematology Am Soc Hematol Educ Program 2003 82 101 14633778

[B13] Asou N Adachi K Analysis of prognostic factors in newly diagnosed patients with acute promyelocytic leukemia: the APL92 study of the Japan Adult Leukemia Study Group (JALSG) Cancer Chemother Pharmacol 2001 48 Suppl 1 S65 S71 10.1007/s002800100308 11587370

[B14] Tallman MS Lefèbvre P Effects of all-trans retinoic acid or chemotherapy on the molecular regulation of systemic blood coagulation and fibrinolysis in patients with acute promyelocytic leukemia J Thromb Haemost 2004 2 1341 1350 10.1111/j.1538-7836.2004.00787.x 15304040

[B15] Kantarjian HM Keating MJ Acute procyelocytic leukemia: MD Anderson Hospital experience Am J Med 1986 80 789 797 10.1016/0002-9343(86)90617-0 3458366

